# FruitSeg30_Segmentation dataset & mask annotations: A novel dataset for diverse fruit segmentation and classification

**DOI:** 10.1016/j.dib.2024.110821

**Published:** 2024-08-10

**Authors:** F.M. Javed Mehedi Shamrat, Rashiduzzaman Shakil, Mohd Yamani Idna Idris, Bonna Akter, Xujuan Zhou

**Affiliations:** a Department of Computer System and Technology, Universiti Malaya, Kuala Lumpur 50603, Malaysia; b Department of Computer Science and Engineering, Daffodil International University, Daffodil Smart City (DSC), Birulia, Savar, Dhaka 1216, Bangladesh; c School of Business, University of Southern Queensland, Springfield, Australia

**Keywords:** Fruit segmentation, Deep learning, Image classification, Dataset diversity, Data annotation, Computer vision, Fruit image, Agriculture automation

## Abstract

Fruits are mature ovaries of flowering plants that are integral to human diets, providing essential nutrients such as vitamins, minerals, fiber and antioxidants that are crucial for health and disease prevention. Accurate classification and segmentation of fruits are crucial in the agricultural sector for enhancing the efficiency of sorting and quality control processes, which significantly benefit automated systems by reducing labor costs and improving product consistency. This paper introduces the “FruitSeg30_Segmentation Dataset & Mask Annotations”, a novel dataset designed to advance the capability of deep learning models in fruit segmentation and classification. Comprising 1969 high-quality images across 30 distinct fruit classes, this dataset provides diverse visuals essential for a robust model. Utilizing a U-Net architecture, the model trained on this dataset achieved training accuracy of 94.72 %, validation accuracy of 92.57 %, precision of 94 %, recall of 91 %, f1-score of 92.5 %, IoU score of 86 %, and maximum dice score of 0.9472, demonstrating superior performance in segmentation tasks. The FruitSeg30 dataset fills a critical gap and sets new standards in dataset quality and diversity, enhancing agricultural technology and food industry applications.

Specification Table

**Table:** 

Subject	Computer Vision and Image Processing
Specific subject area	Automatic Fruit Recognition and Classification, Agricultural Quality Control, Computer Vision, Machine Learning, Deep learning
Type of Data	Raw: Fruits Images (512 × 512 pixels, JPG format) Raw: Segmentation Masks (512 × 512 pixels, PNG format)
Data Collection	The dataset includes high-resolution images of various fruits, segmented into 30 distinct classes. The archive contains 1969 mages entirely, along with their corresponding segmentation masks. Images were captured using various phone cameras in Malaysia, Bangladesh, and Australia under diverse conditions. Each class is organized into two subfolders: “Images” (JPG) and “Mask” (PNG).
Data Source Location	1. Location: USJ 19 City: Subang Jaya Country: Malaysia 2. Location: Mirpur-1, Mirpur-10 City: Dhaka Country: Bangladesh 3. City: Springfield State: Queensland Country: Australia
Data Accessibility	Repository name: Mendeley Data Data identification number: 10.17632/vkht8pfsp3.1 Direct URL to data: https://data.mendeley.com/datasets/vkht8pfsp3/3

## 1 Value of the Data

• *Automated Quality Assessment:* In the agriculture sector, the precise annotations and diverse image collection of the FruitSeg30 dataset enable the development of advanced deep-learning models for automated fruit quality assessment. These models can be integrated into sorting and grading systems in processing facilities to classify fruits based on quality metrics, leading to a more efficient packing and distribution process.
• *Yield Prediction:* Agronomists and agricultural researchers can leverage the dataset to train models capable of predicting fruit yield images captured throughout the growing season. Such predictive models assist in estimating yield quantities, facilitating better resource management and crop planning, thus optimizing agricultural output.
• *Nutritional Monitoring and Diet Management:* The dataset can be utilized by developers of health and wellness applications to recognize and classify fruits in meal photos, providing users with instant nutritional information, including calorie estimates. The application supports monitoring management and encourages healthier eating habits.
• *Educational Tools:* Educational institutions and software developers can use the FRuitSeg30 dataset to create tools and applications that educate students and aspiring professionals in image processing, machine learning and segmentation techniques. These teaching technologies have the potential to greatly improve learning experiences by offering practical exercises with authentic data.
• *Market Analysis and Trend Prediction:* The dataset's comprehensive coverage of fruit varieties across different geographical regions provides valuable insights for market analysis within the agricultural sector. Businesses can use these insights to forecast market trends, plan crop production, and strategize on distribution to meet regional demands effectively.

## 2 Background

The advent of deep learning has significantly enhanced the capabilities of image recognition systems, particularly in the agricultural sector, where such advancements can lead to improved outcomes in areas like yield estimation, fruit sorting, and disease detection [[1], [2], [3], [4], [5]]. Despite these advancements, the performance of machine learning models heavily relies on the quality and diversity of the datasets used during the training process [[6], [7], [8]]. Traditionally, fruit recognition systems have employed datasets captured under controlled environmental conditions, involving fruits presented against uniform backgrounds and consistent lighting conditions [9]. While this simplifies the image segmentation and recognition task, it does not adequately prepare models for the complexities and variabilities in natural environments [10].

Current datasets typically lack the variability necessary to mimic real-world conditions, where fruits appear in diverse settings, often under varying lighting conditions and backgrounds [11]. Furthermore, these datasets rarely include a wide range of fruit types, further limiting the robustness and applicability of the resulting models [12].

The “FruitSeg30_Segmentation Dataset & Mask Annotations” was developed to address these shortcomings by providing a richly annotated dataset captured from a variety of natural and uncontrolled environments across Malaysia, Bangladesh and Australia. This dataset comprises 1969 images across 30 distinct fruit classes, each annotated with precise segmentation masks. This dataset not only advances the field by filling the gap in available resources but also supports the development of more sophisticated image segmentation models that are crucial for applications such as automated fruit quality assessment, yield prediction, and real-time monitoring of fruit growth and health. Additionally, the diverse backgrounds and lighting conditions included in the dataset challenge existing models, pushing the envelope on what these algorithms can achieve and where they fail, thereby providing a path toward significant improvements in model accuracy and reliability.

## 3 Data Description

The “FruitSeg30_Segmentation Dataset & Mask Annotations” consists of a comprehensive collection of high-resolution fruit images meticulously annotated with segmentation masks. This dataset is segmented into 30 distinct fruit classes, providing a total of 1969 images alongside their corresponding segmentation masks. Each image and mask pair has 512 × 512 pixel resolution, ensuring uniformity and high quality for detailed image processing tasks.

The images in this dataset were captured under diverse environmental conditions in Malaysia, Bangladesh, and Australia, ensuring variability in lighting, angles, and backgrounds. This diversity enhances the robustness of the dataset, making it suitable for training and evaluating image segmentation models in real-world scenarios.

Each class folder is organized into two subfolders:

• *Images:* This subfolder contains high-quality JPG images of fruits. The images feature various backgrounds, ranging from natural outdoor settings (with natural lighting and real-world elements) to controlled indoor environments (with consistent lighting and minimal distraction). This variety includes different textures, lighting conditions, and scenes, providing a comprehensive training ground for machine-learning models.
• *Mask:* This subfolder contains the corresponding segmentation masks in PNG format. These masks are binary images where the fruit is clearly delineated from the background. In these masks, white pixels represent the fruit, while black pixels represent the background. The masks provide a precise annotation, which is essential for accurate segmentation tasks.

### 3.1 Technical details

**Fig. 1:**
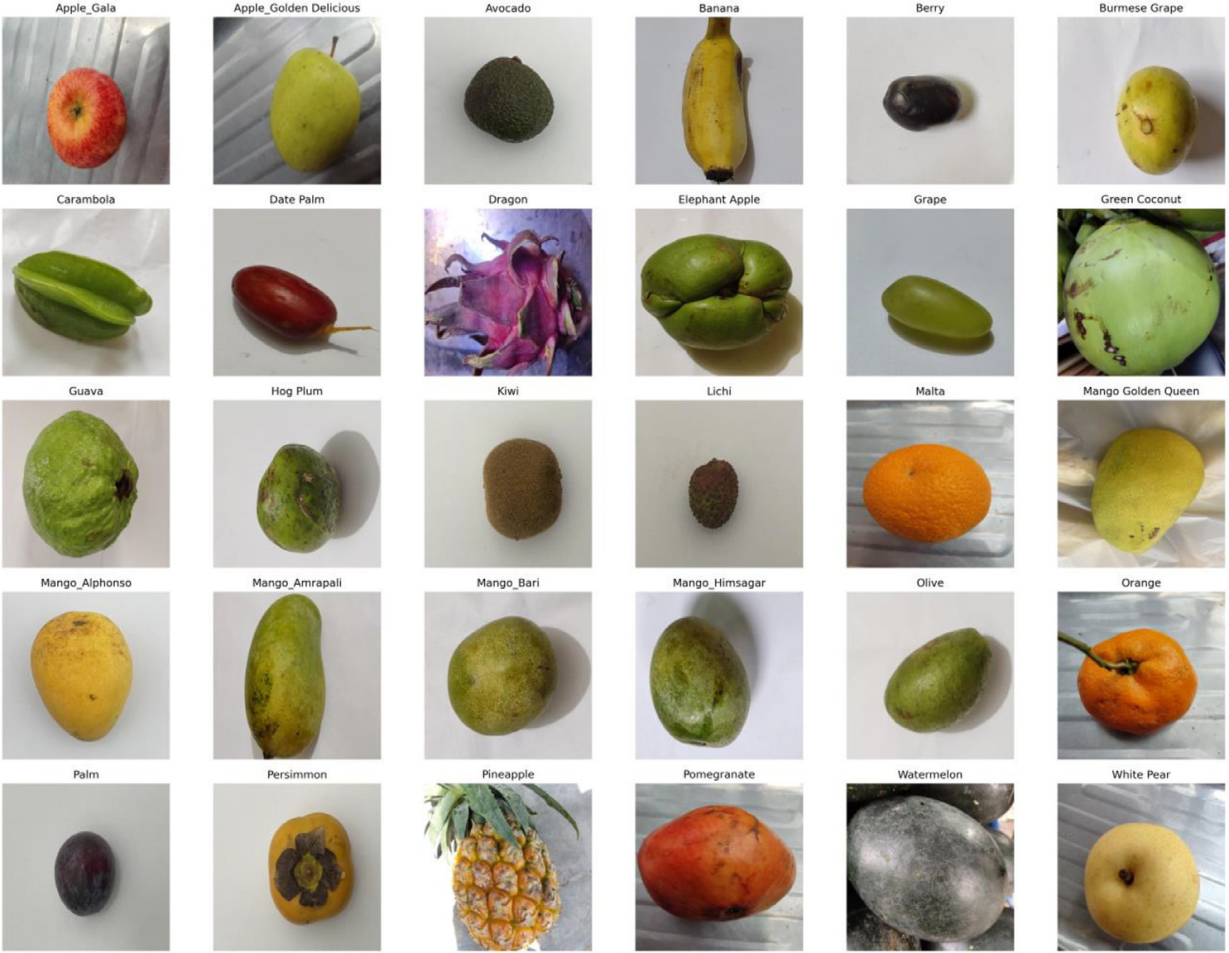
Representative images from each of the 30 classes in the FruitSeg30 dataset.

• *Image Resolution and Quality:* Each image in the dataset is 512 × 512 pixels, a resolution that captures fine details necessary for high-precision segmentation. The high resolution supports detailed feature extraction and model training.
• *Lighting and Angle Variations:* The dataset includes images taken under various lighting conditions, from direct sunlight to shaded environments, and from multiple angles to ensure that models trained on this data can handle real-world variability.
• *Background Complexity:* The dataset includes images with varying degrees of background complexity, from plain, uncluttered backgrounds to complex, textured ones. This variability helps develop models that can generalize well to different real-world conditions.
• *Class diversity:* The dataset comprises 30 different fruit classes covering various fruit types. The classes are: ‘Apple_Gala’, ‘Apple_Golden Delicious’, ‘Avocado’, ‘Banana’, ‘Berry’, ‘Burmese Grape’, ‘Carambola’, ‘Date Palm’, ‘Dragon’, ‘Elephant Apple’, ‘Grape’, ‘Green Coconut’, ‘Guava’, ‘Hog Plum’, ‘Kiwi’, ‘Lichi’, ‘Malta’, ‘Mango Golden Queen’, ‘Mango_Alphonso’, ‘Mango_Amrapali’, ‘Mango_Bari’, ‘Mango_Himsagar’, ‘Olive’, ‘Orange’, ‘Palm’, ‘Persimmon’, ‘Pineapple’, ‘Pomegranate’, ‘Watermelon’, ‘White Pear’. This diversity enhances the dataset's applicability across various fruit recognition and segmentation tasks. Fig. 1 illustrates the sample images of each class from the dataset.

### 3.2 Data structure

**Fig. 2:**
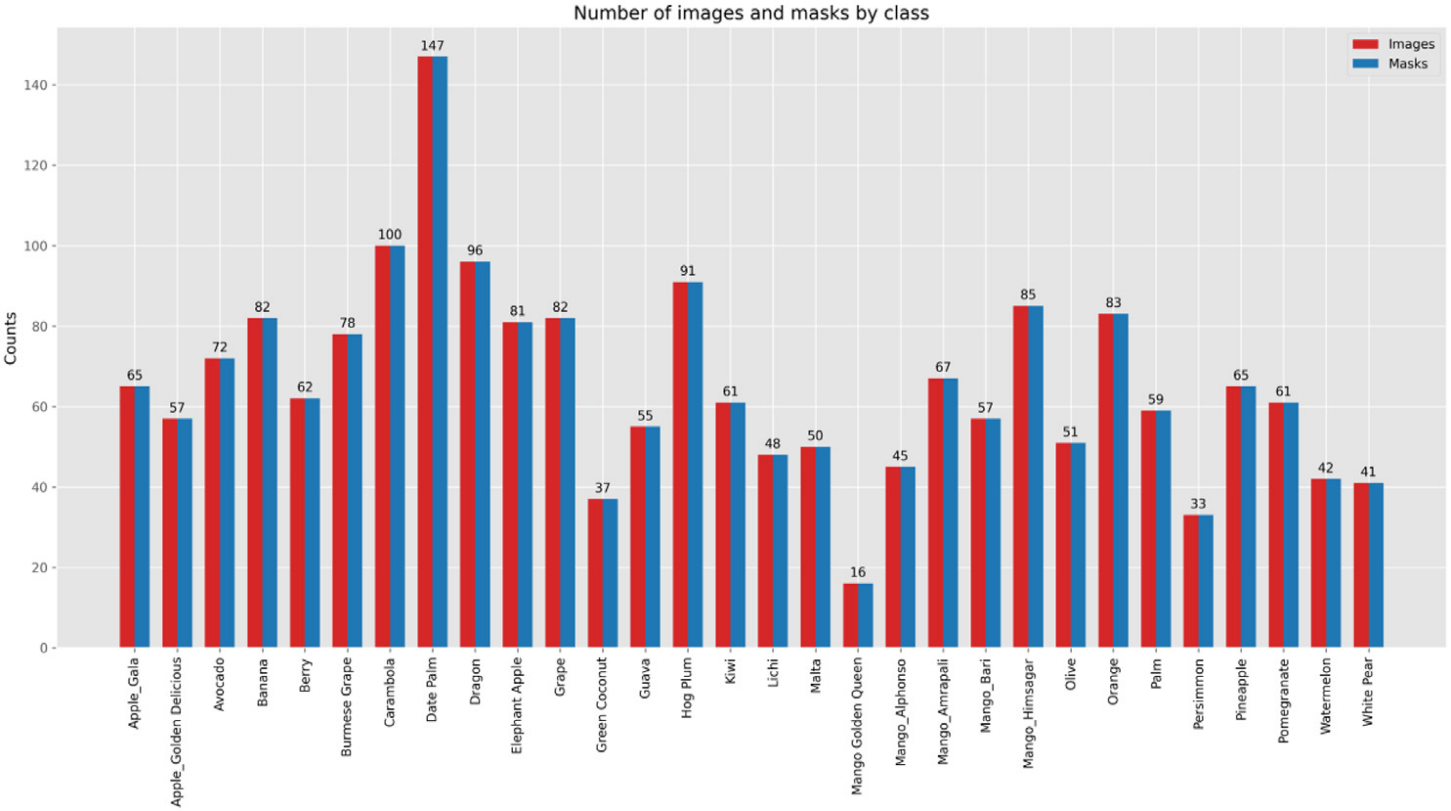
Provide an overview of the dataset's number of images and segmentation masks.

• *Organized Directory Structure:* The dataset is nearly organized into directories for each fruit class, with subdirectories for images and masks. The structure facilitates easy access and efficient use of datasets for various image processing tasks.
• *File Naming Convention:* Consistent file naming conventions are used across the dataset to ensure that corresponding images and masks can be easily matched. For each class, images are named as 1.jpg, 2.jpg, etc., and their corresponding masks are named as 1_mask.png, 2_mask.png, etc. This consistency is critical for training and validating machine learning models effectively. Fig. 2 demonstrates a detailed overview of the dataset.

## 4 Experimental Design, Materials and Methods

### 4.1 Data collection techniques

• *Capture Devices:* Images were taken using various cameras to capture a broad spectrum of lighting conditions, angles, and backgrounds. This approach ensures a realistic representation of fruits in different environments.
• *Environmental Conditions:* Images were captured in both natural and controlled settings, reflecting a wide array of scenarios that fruits may be subjected to in real-world conditions. This includes different times of day, verifying light intensities, and diverse geographical locations.
• *Segmentation Masks:* The mask creation process for the “FruitSeg30_Segmentation Dataset & Mask Annotations” dataset involves automated background removal and manual verification to ensure high accuracy and reliability. The primary tool used for this process is the ‘rembg’ library, which leverages advanced machine learning techniques to separate the foreground (fruit) from the background, and create precise binary masks. Each image I is loaded using the Python Imaging Library (PIL).
  (1) I=PIL.Image.open(input_path)

The ‘rembg’ library is applied to remove the background, producing an image $I_{no\_bg}$ with the fruit in the foreground and a transparent background.

(2) $$I_{no\_bg}=rembg.remove\;\left(I\right)$$

A binary mask M is created from the processed image. The mask is a grayscale image where the fruit is represented by white pixels (value 255) and the background by black pixels (value 0). This is achieved by extracting the alpha channel α of $I_{no\_bg}$.

(3) $$M\left(x,y\right)=\;\{\begin{array}{c} 255\\ 0 \end{array}\;\begin{array}{c} if\;\alpha \;\left(x,y\right)>0\\ otherwise \end{array}$$

Then, the processed image $I_{no\_bg}$ and the binary mask M are saved in the respective directories. The entire process of generating segmented masks is illustrated in Algorithm 1.

**Table:** **Algorithm 1**: Mask creation for FruitSeg30 dataset.

**Require:** Input directory: $D_{input}$, Output directory: $D_{output}$, Mask directory: $D_{mask}$
**Expected Output:** Processed images and binary masks
1: Create $D_{output}$ and $D_{mask}$ if they do not exist
2: **for** each file f in $D_{input}$**do**
3: **if**f ends with ‘.png’, ‘.jpg’, or ‘.jpeg’ **then**
4: Load image I←PIL.Image.open(f)
5: Remove background $I_{no\_bg}$← rembg. remove (I)
6: Save $I_{no\_bg}$ to $D_{output}$ as PNG
7: Create binary mask M← Image.new (“*L*”, $I_{no\_bg}$.size, 0)
8: Extract alpha channel α$\leftarrow \;I_{no\_bg}$.getchannel (‘*A*’)
9: **for** each pixel (x,y) in α**do**
10: **if**α(x,y)>0**then**
11: M(x,y)← 255
12: **else**
13: M(x,y)← 0
14: **end if**
15: **end for**
16: **Save**M to $D_{mask}$ as PNG
17: **end if**
18: **end for**

### 4.2 Preprocessing

**Fig. 3:**
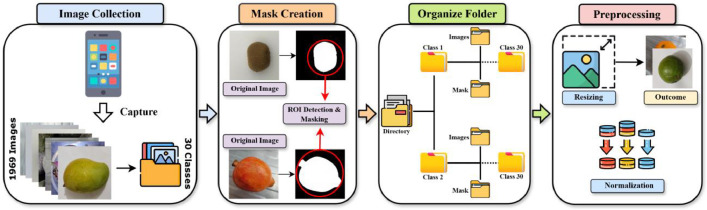
Entire pipeline of dataset collection and processing.

The “FruitSeg30_Segmentation Dataset & Mask Annotations” dataset comprises a meticulously curated collection of 1969 images across 30 distinct fruit classes, selected from various environments to ensure a representative and diverse sample for the purpose of training a deep learning model. Each image and its corresponding mask were resized to a consistent resolution of 512×512 pixels to standardize input processing across the dataset. Furthermore, the pixel values were normalized to the range [0, 1] by dividing by 255, ensuring data scaling and improving deep learning model training consistency. Fig. 3 represents the entire procedure of the dataset collection and processing.

### 4.3 Segmentation using U-Net

The U-Net model [13] employed for segmentation consists of an encoder-decoder architecture with skip connections. The encoder (contracting path) reduces the spatial dimensions while increasing the feature dimensions, and the decoder (expansive path) progressively recovers the spatial resolution and detail.

#### 4.3.1 U-Net architecture

• *Encoder:* The contracting path includes four levels of double conventional layers followed by a max-pooling layer. Each convolutional layer uses a 3 × 3 kernel with ReLU activation. The sequence of feature channels across the encoder is as follows: 64 → 128 → 256 → 512. Max-pooling is performed with a 2 × 2 window and stride of 2, reducing the spatial dimensions by half after each level.
• *Bottleneck:* At the bottom of the U-Net, a bridge connects the contracting and expansive paths, consisting of two 3 × 3 convolutions with 1024 filters each, followed by ReLU activation. This section is crucial as it processes the most compressed feature representation.
• *Decoder:* The expansive path mirrors the encoder, using transposed convolutions for upsampling. At each stage, the feature map is upsampled, followed by a concatenation with the corresponding cropped feature map from the encoder path, maintaining high-resolution features for precise localization. The layers in the decoder are structured as follows: 512 → 256 → 128 → 64.

#### 4.3.2 Loss function and optimization

The model optimization was performed using the Adam optimizer [14] with a learning rate of 1 × 10 ^−4^. A custom dice loss [15] function was utilized, defined as:

(4) $$L\;\left(y_{true},\;y_{pred}\right)\;=\;1\;-\;\frac{2.\sum \left(y_{true}.y_{pred}\right)+\;1}{\sum y_{true}+\;\sum y_{pred}+\;1}$$

This loss function is particularly effective for data with imbalanced classes, as it helps achieve a balance between precision and recall by maximizing the overlap between predicted and true masks. Algorithm 2 provides a detailed overview of the applied U-Net model.

**Table:** **Algorithm 2:** Applied U-Net Architecture.

**Require:** Input image I of shape (H,W,C).
**Ensure:** Segmented output image O of shape (H,W,C)
1: **Encoder:**
2: **for** level l=1 to 4 **do**
3: Apply double 3 × 3 convolutional layers with ReLU activation: $C_{l\;}=$ Conv3×3(ReLU(Conv3×3 (I)))
4: Apply max-pooling with 2 × 2 window and stride of 2: $P_{l\;}=$ Maxpool2×2 ($C_{l\;}$)
5: Increase the number of feature channels: $C_{l+1\;}=\;2\times C_{l\;}$
6: **end for**
7: **Bottleneck:**
8: Apply double 3 × 3 convolutional layers with 1024 filters and ReLU activation: $B=\text{Conv}3\times 3(\text{ReLU}(\text{Conv}3\times 3(P_{4\;})))$
9: **Decoder:**
10: **for** level l=4 to 1 **do**
11: Apply transposed convolution for upsampling: $U_{l}=\;UpConv\;\left(B\right)$
12: Concatenate with corresponding feature map from encoder: $M_{l\;}=\;Concat\left(U_{l},\;C_{l}\right)$
13: Apply double 3 × 3 convolutional layers with ReLU activation: $D_{l}\;=\;\text{Conv}3\times 3\left(\text{ReLU}(\text{Conv}3\times 3(M_{1}))\right)$
14: Decrease the number of feature channels: $D_{l-1}=$$\frac{D_{l}}{2}$
15: **end for**
16: **Output Layer:**
17: Apply 1 × 1 convolution to map to output channels: $O\;=\;Conv1\times 1\;(D_{l})$
18: **Loss Function and Optimization:**
19: Define custom dice loss function: $L\;\left(y_{true\;},\;y_{pred}\right)\;=\;1\;-\;\frac{2.\sum (y_{true}.y_{pred})+\;1}{\sum y_{true}+\;\sum y_{pred}+\;1}$
20 Optimize using Adam optimizer with learning rate 1 × 10^−4^

#### 4.3.3 Training, validation and analysis

The U-Net model was methodically trained over 100 epochs using a batch size of 8. Several strategies were employed to mitigate the risk of overfitting, which was particularly important due to the model's complexity and the detailed nature of image segmentation tasks. Callbacks for early stopping were set to monitor the validation loss, terminating training if the loss did not improve for a specified number of epochs. This approach ensures that the model retains its generalization capabilities and does not merely memorize the training data. Additionally, a model checkpoint strategy was implemented to save the model weights at the epoch where it achieved the lowest validation loss, thereby capturing the most effective version of the model during the training process.

**Fig. 4:**
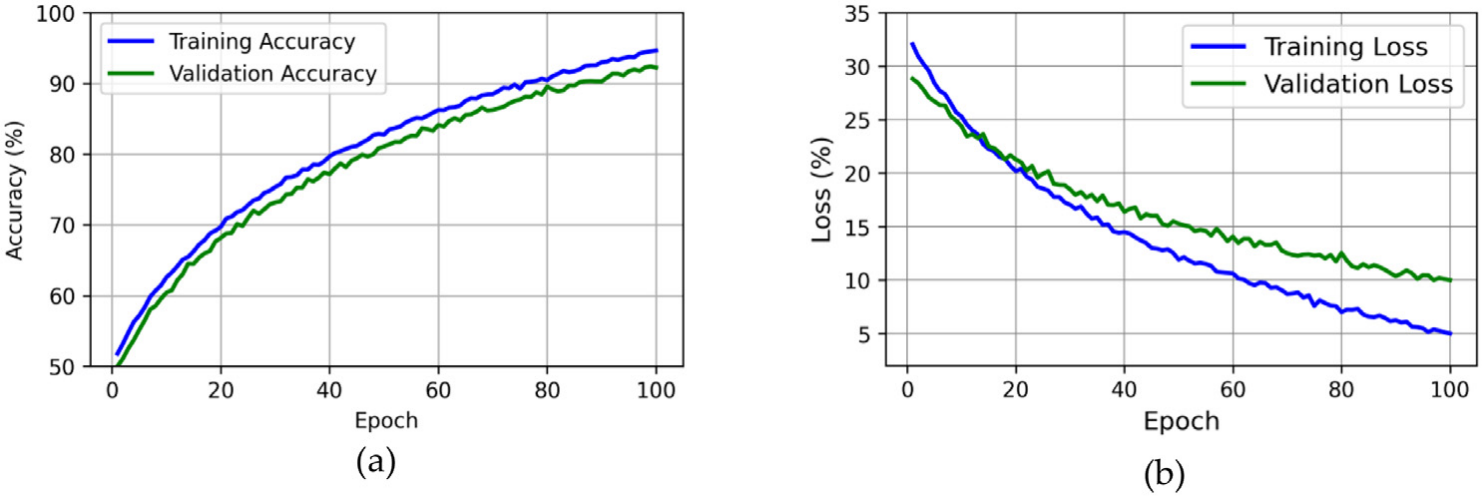
Performance metrics over 100 epochs, showing (a) training and validation accuracy and (b) training and validation loss.

Fig. 4 illustrates the performance metrics for the model's training and validation phases over 100 epochs. In Fig. 4(a), the training accuracy (blue line) and validation accuracy (green line) demonstrate a consistent upward trend, including that the model is learning effectively throughout the training process. The training accuracy starts at approximately 50 % and steadily increases to over 94 % by the 100th epoch. Similarly, the validation accuracy begins at around 48 % and rises to 92.5 %. The alignment of the training and validation accuracy curves indicates that the model is generalizing well to the validation data, with no significant overfitting. Subsequently, Fig. 4(b) depicts the training and validation loss curves. The training loss (blue line) starts at a higher value of 33 % and decreases steadily to around 5 % by the end of the training period. The validation loss (green line) follows a similar trend, starting at 30 % and decreasing to about 10 %. The decreasing loss curves indicate that the model's predictions are becoming more accurate over time, and the small gap between the training and validation loss further confirms the model's good generalization capabilities. The stability of the dataset contributes significantly to the model's accuracy, as consistent and representative data ensures the reliability and robustness of the training process.

Table 1 provides the summary of the performance of the applied U-Net model on our dataset “FruitSeg30_Segmentation Dataset & Mask Annotations”. The model processes images with an input specification of (256, 256, 3), enabling detailed feature extraction crucial for precise image segmentation. Over 1579 samples were trained with an 80:20 train/validation split over 100 epochs, achieving an outstanding training and validation accuracy of 94.72 % and 92.57 %, respectively. The model's precision was 94 %, recall 91 %, F1 score 92.5 %, and IoU scores 86 %, indicating high accuracy and balanced performance in segmentation tasks. These impressive metrics highlight the robustness and effectiveness of the “FruitSeg30” dataset, which includes 30 distinct fruit classes with comprehensive segmentation masks.

**Table 1:** Parameters and performance summary of the U-Net model.

Model	Input Specification	Number of Training Samples	Train/ Val Split	Epochs	Training Accuracy	Validation Accuracy	Training Time	Precision	Recall	F1-Score	IoU Score
U-Net	(256, 256, 3)	1579	80:20	100	94.72 %	92.57 %	6055s	94 %	91 %	92.5 %	86 %

Furthermore, the outputs (Table 2) from the U-Net model applied to our dataset demonstrate a broad spectrum of segmentation capabilities, with varying Dice Scores across different fruit types that underscore the dataset's robustness and diversity. The scores highlight the dataset's correctness in presenting challenging scenarios involving reflective surfaces and complex textures, providing invaluable opportunities for rigorous model testing and enhancement. The scores also illustrate the dataset's comprehensive coverage of textural and color complexities, making it an excellent tool for advancing segmentation techniques across varied conditions. This dataset not only includes a wide array of fruit types, each presenting a unique challenge in terms of shape, texture, and background conditions, but it also enriches the field of machine learning by introducing real-world complexities that are crucial for developing robust models.

**Table 2:** Overview of U-Net outputs displaying original mage with true mask, predicted segmented mage, and dice scores.

#### 4.3.4 Confusion matrix analysis

**Fig. 5:**
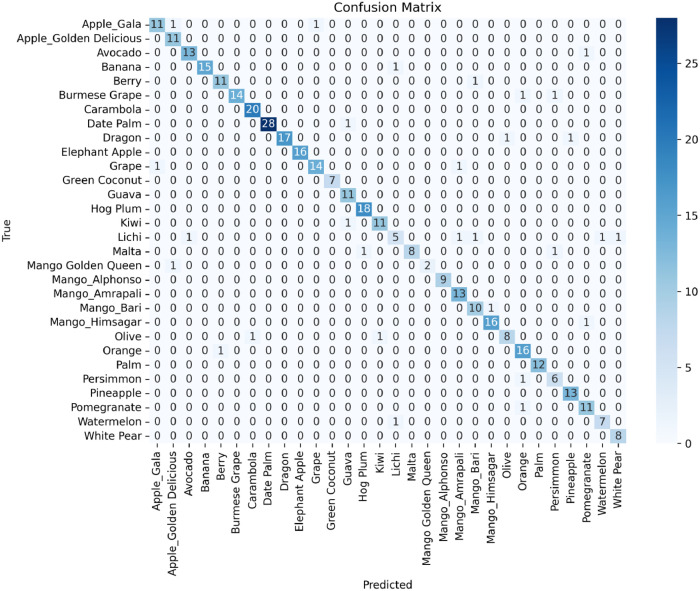
Confusion matrix illustrating the U-Net model's performance on the validation set of the dataset, highlighting correct classifications and misclassifications across 30 fruit classes.

The confusion matrix presented in Fig. 5 provides a detailed analysis of the U-Net model's performance on the validation set of the dataset, which comprises 390 images across 30 different fruit classes. Each cell in the matrix represents the number of instances where the actual class (True label) matches the predicted class, with diagonal elements indicating correct predictions and off-diagonal elements indicating misclassifications.

The diagonal elements, which represent the correct classifications for each fruit class, show high values, reflecting the model's high accuracy. For instance, the model correctly identified out of 13 Apple_Gala images, 11 out of 11 Apple_Golden Delicious images, and 13 out of 14 Avocado images. Similar high accuracies are observed across most classes, highlighting the model's robustness and reliability. Misclassification, represented by the off-diagonal elements, is relatively sparse, indicating that the model rarely confuses one fruit class with another. For example, there is only one misclassification for Apple_Gala, Avocado, Date Palm, and two in Olive, which was incorrectly predicted as another class. Such low misclassification rates further underline the effectiveness of the FruitSeg30 dataset in training accurate segmentation models.

The confusion matrix also emphasizes the model's ability to generalize well across diverse fruit classes. This strong performance can be attributed to the high-quality annotations and diverse environmental conditions captured in the dataset, which include variations in lighting, angles and backgrounds.

#### 4.3.5 Hardware and software

Experiments were conducted on a computing system equipped with a Ryzen 7 3800X processor, 32GB of RAM, and an NVIDIA RTX 4070 GPU. The software stack was managed via Anaconda, facilitating an organized package management and deployment environment. The model was developed and tested using Spyder IDE, part of the Anaconda Suite, which provided an efficient and user-friendly interface for writing and debugging Python code. The model was implemented using TensorFlow 2.x and Keras, providing a flexible and powerful platform for designing and training deep learning models.

## 5 Analysis of the Model Performance and Dataset Characteristics

The comparative analysis in Table 3 demonstrates the robustness and high performance of the U-Net model trained on the FruitSeg30 dataset. The U-Net model achieved an accuracy of 92.57 %, precision of 94 %, recall of 91, F1-score of 92.5 % and an IoU score of 86 %. These metrics are competitive and often surpass those achieved with other datasets. For instance, M.D. Barbole et al. [25] reported a precision of 95 % and an F1-score of 90.28 % using the U-Net model on the Embrapa Wine Grape Instance Segmentation Dataset (WGISD). In comparison, our study achieved a higher F1-score of 92.5 % and comparable precision of 94 % using the U-Net model on our novel dataset. Similarly, the IoU score of 86 % on the FruitSeg30 dataset is significantly higher than the 71.6 % reported by X. Ni et al. [27] using Mask R-CNN on the Blueberry traits extraction and analysis dataset. Additionally, the comparison shows that while the Total generalized variation fuzzy C means (TGVFCMS) model by V. G Krishnan et al. [26] achieved a high accuracy of 93.45 %, it lacks reported precision, F1-score, and IoU, making it difficult to assess the overall segmentation performance comprehensively. In contrast, the FruitSeg30 dataset's comprehensive metrics provide a clearer view of its effectiveness.

**Table 3:** Comparison of the model's performance on the FruitSeg30 dataset with other existing datasets.

Authors Name	Dataset Name	Model	Accuracy (%)	Precision (%)	Recall (%)	F1-score (%)	Best Dice Score (%)	IoU Score (%)
K Kestur [24]	MangoNet-Semantic-Dataset	CNN	73.6	–	–	84.4	–	–
M. D. Barbole et al. [25]	Embrapa Wine Grape Instance Segmentation Dataset (WGISD)	U-Net	89	95	86	90.28	–	–
V. G Krishnan et al. [26]	Real Dataset of Bananas in CIAT's image library	Total generalized variation fuzzy C means (TGVFCMS)	93.45	–	89.04	–	–	–
X. Ni et al. [27]	Blueberry traits extraction and analysis	Mask R-CNN	90.4	–	–	–	–	71.6
G Lin et al. [28]	Guava dataset with 304 RGB-D	Tiny Mask R-CNN	–	53.7	52.3	51.5	–	50
B. A. Farisqi and A. Prahara [29]	Locally collected	Mask R-CNN	–	90	88	89	–	–
S. Abinaya et al. [30]	Plant village dataset	U-Net	90.74	–	–	51.70	54.25	62.85
S. Mane et al. [31]	Locally collected	U-Net	–	93.27	86.79	89.91	–	–
K. Sun et al. [32]	COCO-Stuff dataset	DeepLab-ResNet	–	87.4	72.7	79.6	–	65.3
**Shamrat et al.**	**FruitSeg30_Segmentation Dataset & Mask Annotations**	**U-Net**	**92.57**	**94**	**91**	**92.5**	**94.72**	**86**

Table 4 provides a comprehensive analysis of the “FruitSeg30_Segmentation Dataset & Mask Annotations” in relation to the other datasets within the field, highlighting its distinctive attributes and strengths. This dataset stands out significantly compared to existing collections due to its diverse geographical coverage, extensive class variety, and superior annotation quality. It is distinguished from other datasets by its international scope, which further enhances its robustness and applicability in various global contexts, a critical factor in the development of universally effective machine learning models. Although it comprises 1969 images, which may seem modest relative to larger datasets, it uniquely covers 30 classes, thereby offering a broader range of categories for more comprehensive image analysis tasks. A distinguishing feature of this dataset is the inclusion of segmentation masks, which are invaluable for precise image segmentation tasks and are only paralleled by a few other datasets. The images are also provided at a resolution of 512×512 pixels, which is particularly suitable for practical machine learning applications that require moderate image detail without the computational burden associated with higher resolutions. This resolution balances the need for details with computational efficiency. Consequently, this dataset not only satisfies but also surpasses the convolutional standards for dataset construction, providing a versatile instrument for advancing research in image processing and machine learning in various realistic environments.

**Table 4:** A comparative analysis of the related datasets based on various properties.

Authors	No. of Images	Classes	Image Format	Geographical Diversity (International)	Segmentation Mask	Resolution	Lighting Conditions	Background Complexity	Capture Protocols	Annotation Process	Environment
B. Pakruddin and R. Hemavathy [16]	5009	5	JPG	✗	✗	3120 × 3120	Diverse	Complex	Distances	✗	Natural and controlled
J. Gené-Mola et al. [17]	3925	5	JPG	✗	✓	1024 × 1024	Diverse	Complex	Distances	Automated	Natural
P. Pathmanaban et al. [18]	2095	3	JPG	✗	✗	3000 × 300	Uniform	Plain	Single Angle	✗	Controlled
A. K. Maitlo et al. [19]	2309	3	JPG	✗	✗	850 × 1300	Uniform	Plain	Single Angle	✗	Controlled
S. I. Ahmed et al. [20]	1800	7	PNG	✗	✗	240 × 320	Diverse	Plain	Multiple Angles, Distances	✗	Controlled
A. Rajbongshi et al. [21]	681	6	JPG	✗	✓	512 × 512	Diverse	Complex	Multiple Angles, Distances	Manual	Natural
M. R. Sheikh et al. [22]	1166	6	JPG	✗	✗	4608 × 3456	Diverse	Complex	Multiple Angles	✗	Controlled
T. Khatun et al. [23]	3779	2	JPG	✗	✗	256 × 256	Diverse	Complex	Multiple Angles	✗	Natural
**Shamrat et al**	**1969**	**30**	**JPG**	✓	✓	**512** × **512**	**Diverse**	**Complex**	**Multiple Angles, Distances**	**Automated + Manual Verification**	**Natural and controlled**

## Limitations

The FruitSeg30 dataset, although characterized by a variety of unique attributes, is limited by its relatively small size, consisting of 1969 images. To maintain the original quality and integrity of the dataset, it was kept in its unmodified, raw form without any augmentation. This limitation may restrict the generalizability of models trained on this dataset. To address this issue, researchers can employ various data augmentation techniques, including rotations, flips, scaling, cropping and color adjustments. These methods help artificially expand the dataset's size. Additionally, synthetic data generation methods such as Generative Adversarial Networks (GANs) can be used to create more training samples. Despite its limitations, the dataset's high quality, detailed annotations and diverse environmental conditions significantly enhance its robustness and applicability for fruit segmentation tasks.

## Ethics Statement

This article does not involve any research on human or animal subjects by any authors. The datasets used are publicly accessible. It is essential to adhere to established citation guidelines when employing the datasets.

## CRediT Author Statement

**F.M. Javed Mehedi Shamrat:** Conceptualization, Methodology, Software, Validation, Formal analysis, Investigation, Resources, Data curation, Writing—original draft preparation, writing—review and editing, visualization; **Rashiduzzaman Shakil:** Investigation, Software, Validation, Formal analysis, Resources, Writing—original draft preparation; **Mohd Yamani Idna Idris:** Validation, Formal analysis, writing—review and editing, Supervision; **Bonna Akter:** Resources, Software, Formal analysis, visualization; **Xujuan Zhou:** Resources, writing—review and editing, Supervision.

## Data Availability

FruitSeg30_Segmentation Dataset & Mask Annotations (Original data) (Mendeley Data).
